# Novel insights into ferroptosis: Implications for age-related diseases

**DOI:** 10.7150/thno.50663

**Published:** 2020-10-26

**Authors:** Ren-Peng Zhou, Yong Chen, Xin Wei, Bin Yu, Zhi-Gang Xiong, Chao Lu, Wei Hu

**Affiliations:** 1 Department of Clinical Pharmacology, The Second Hospital of Anhui Medical University, Hefei 230601, China.; 2 Department of Neurobiology, Morehouse School of Medicine, Atlanta, GA 30310, USA.

**Keywords:** age-related diseases, ferroptosis, lipid peroxidation, iron, reactive oxygen species

## Abstract

Rapid increase in aging populations is an urgent problem because older adults are more likely to suffer from disabilities and age-related diseases (ARDs), burdening healthcare systems and society in general. ARDs are characterized by the progressive deterioration of tissues and organs over time, eventually leading to tissue and organ failure. To date, there are no effective interventions to prevent the progression of ARDs. Hence, there is an urgent need for new treatment strategies. Ferroptosis, an iron-dependent cell death, is linked to normal development and homeostasis. Accumulating evidence, however, has highlighted crucial roles for ferroptosis in ARDs, including neurodegenerative and cardiovascular diseases. In this review, we a) summarize initiation, regulatory mechanisms, and molecular signaling pathways involved in ferroptosis, b) discuss the direct and indirect involvement of the activation and/or inhibition of ferroptosis in the pathogenesis of some important diseases, and c) highlight therapeutic targets relevant for ARDs.

## Introduction

Over the past several decades, human life expectancy has steadily increased due to benefits from nutrition, technological advances, and improvements in medical care and vaccination 1. It is estimated that by 2050, the number of people over the age of 60 will reach 2.1 billion 2. With this steady increase in the number of older adults, age has become a major risk factor for age-related diseases (ARDs). ARDs are characterized by continuous cell loss and deterioration in the quality or function of tissues and organs, resulting in increased susceptibility and vulnerability to certain diseases 3. Without control measures, this inevitably leads to loss of mobility, aggravation of disease processes, and increased mortality coupled with severe economic and societal burdens 4. The etiology and pathogenesis of ARDs are complex and remain unclear, greatly limiting clinical diagnosis and treatment of these diseases. Although undetermined factors cause ARDs, the deleterious and progressive changes in multiple organ systems in most ARDs share some basic mechanistic precepts, including oxidative stress, iron accumulation, inflammation, cell injury, and dysfunction 5-7.

Ferroptosis, an iron-dependent non-apoptotic cell death, can be initiated by small molecules that inhibit glutathione biosynthesis or the glutathione-dependent antioxidant enzyme glutathione peroxidase 4 (GPX4), characterized by the iron-dependent accumulation of reactive oxygen species (ROS) and depletion of plasma membrane polyunsaturated fatty acids (PUFAs) 8, 9. When the intracellular lipid ROS level exceeds the antioxidant activity of GPX4, it leads to the disruption of redox homeostasis 10. Ferrous iron can trigger harmful peroxidation of PUFAs in membrane phospholipids by forming toxic lipid radicals, eventually leading to cell death 11. Iron accumulating within several organs during aging, such as brain and muscle, leads to increased oxidative damage and functional decline. Preclinical Alzheimer's disease patients have the highest iron in the cerebral cortex and cerebellum, accompanied by the gradual impairment of cognitive function, suggesting that an imbalance in iron homeostasis is a precursor of neurodegeneration in Alzheimer's disease 12. Abnormal iron deposition in the substantia nigra, especially in the RII region, can be used as a biomarker to distinguish Parkinson's disease patients from healthy controls and to assess the severity of the disease 13. Several studies also have revealed that iron accumulation contributes to cell death of organs and tissues in the pathogenesis and progression of amyotrophic lateral sclerosis, Huntington's disease, cardiomyopathy and type 1 diabetes, and therefore the level of iron as a biomarker for the potential role of ferroptosis and an important causative factor for ARDs 14-17. Recent advances have identified numerous small molecule ferroptosis inducers and inhibitors, including erastin, glutamate, liproxstatin-1 (Lip-1), and ferrostatin-1 (Fer-1). These compounds have been highly valuable for the study of ferroptosis in different diseases* in vivo* and *in vitro*, and the data indicated that ferroptosis might be a potential novel target for therapeutic intervention in ARDs 18, 19.

Emerging studies have confirmed that ferroptosis contributes substantially to the pathogenesis of a variety of ARDs, including neurodegenerative and cardiovascular diseases (CVDs). Intervention in ferroptosis pathways effectively inhibits the progression of these disorders, suggesting ferroptosis as a potential treatment target for these diseases. Herein, we provide a summary of the current knowledge on the mechanism of ferroptosis, its functional roles in the development of ARDs, and its potential for pharmacological and therapeutic targeting of ARDs.

## Characteristics and mechanisms of ferroptosis

### Ferroptosis represents a non-apoptotic programmed cell death

**Figure 1:**
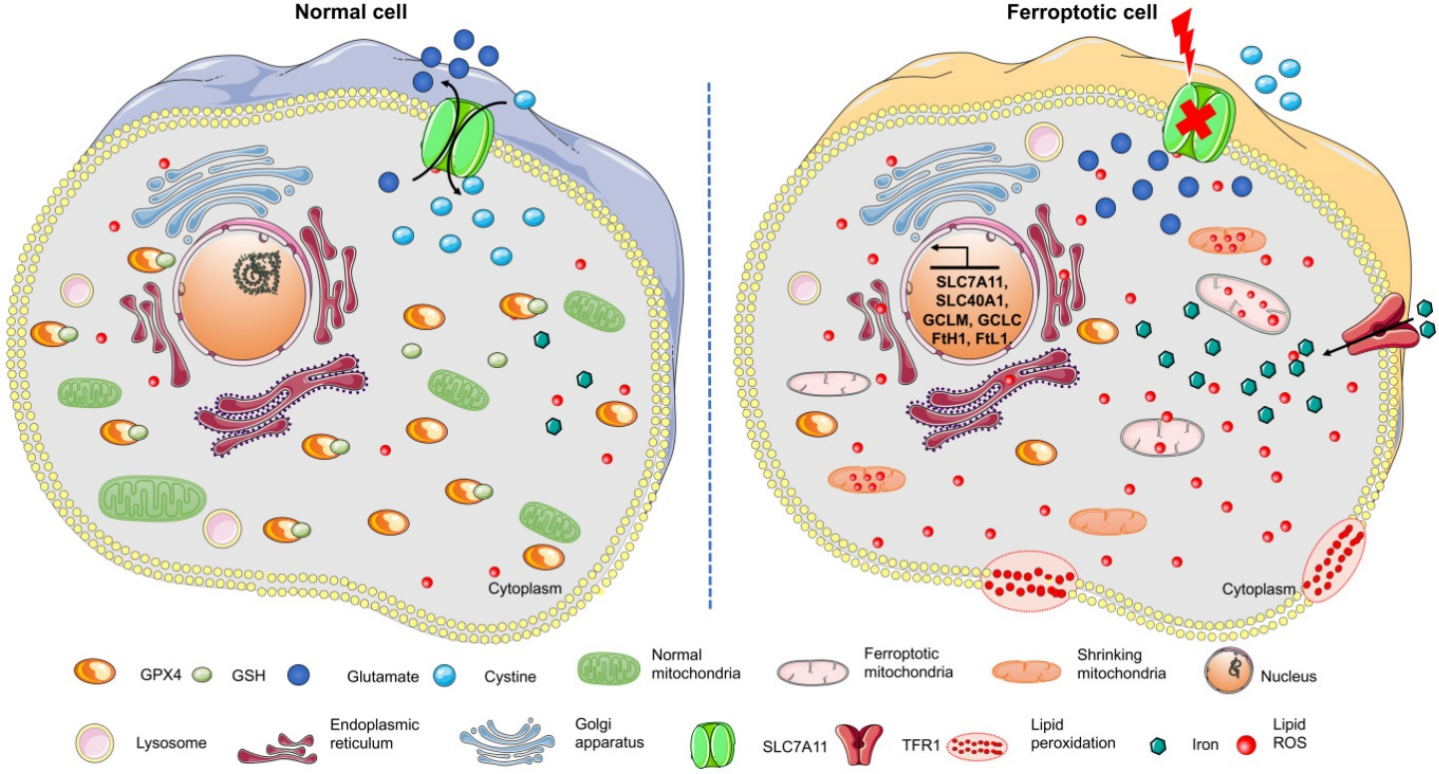
Changes in the morphological and bioenergy characteristics during ferroptosis, including mitochondrial shrinkage, membrane rupture, excess ROS, iron overload, and intracellular GSH depletion. Features of this figure were adapted from Servier Medical Art (http://smart.servier.com/) licensed under a Creative Commons Attribution 3.0 Unported License.

Ferroptosis, a type of cell death induced by erastin or Ras-selective lethal small molecule 3 (RSL3), has attracted much attention in recent years 8. It is an iron-dependent oxidative cell death that plays a special role in lipid peroxide accumulation in the absence of GPX4. Sensitivity to ferroptosis strongly depends on antioxidant metabolism, lipid homeostasis, and the dynamic equilibrium of iron 20. As a different form of programmed cell death, ferroptosis plays a critical role in organismal homeostasis and disease pathologies. Unlike the other known forms of programmed cell death, ferroptosis has unique morphological and bioenergy characteristics, including mitochondrial shrinkage, mitochondrial bilateral membrane thickening and rupture, and intracellular NADPH depletion without altering ATP levels (**Figure 1**). Furthermore, ferroptosis is often accompanied by iron-dependent lipid peroxide accumulation and mitochondrial ROS production. Evidence has confirmed that unlike apoptosis, ferroptosis is not related to caspase activity 21. Also, necroptosis is accompanied by cellular swelling, disruption of plasma membrane integrity, and the release of intracellular contents, while RIP1/RIP3, the key regulators of necroptosis, are not involved in ferroptosis 22. The inhibition of autophagy by 3-MA also does not modulate ferroptosis 8. These findings identify ferroptosis to be a new form of programmed cell death. Given the involvement of ferroptosis in various illnesses, understanding its initiation and underlying regulatory mechanisms may be of great therapeutic significance.

### Ferroptosis is induced by cellular iron

**Figure 2:**
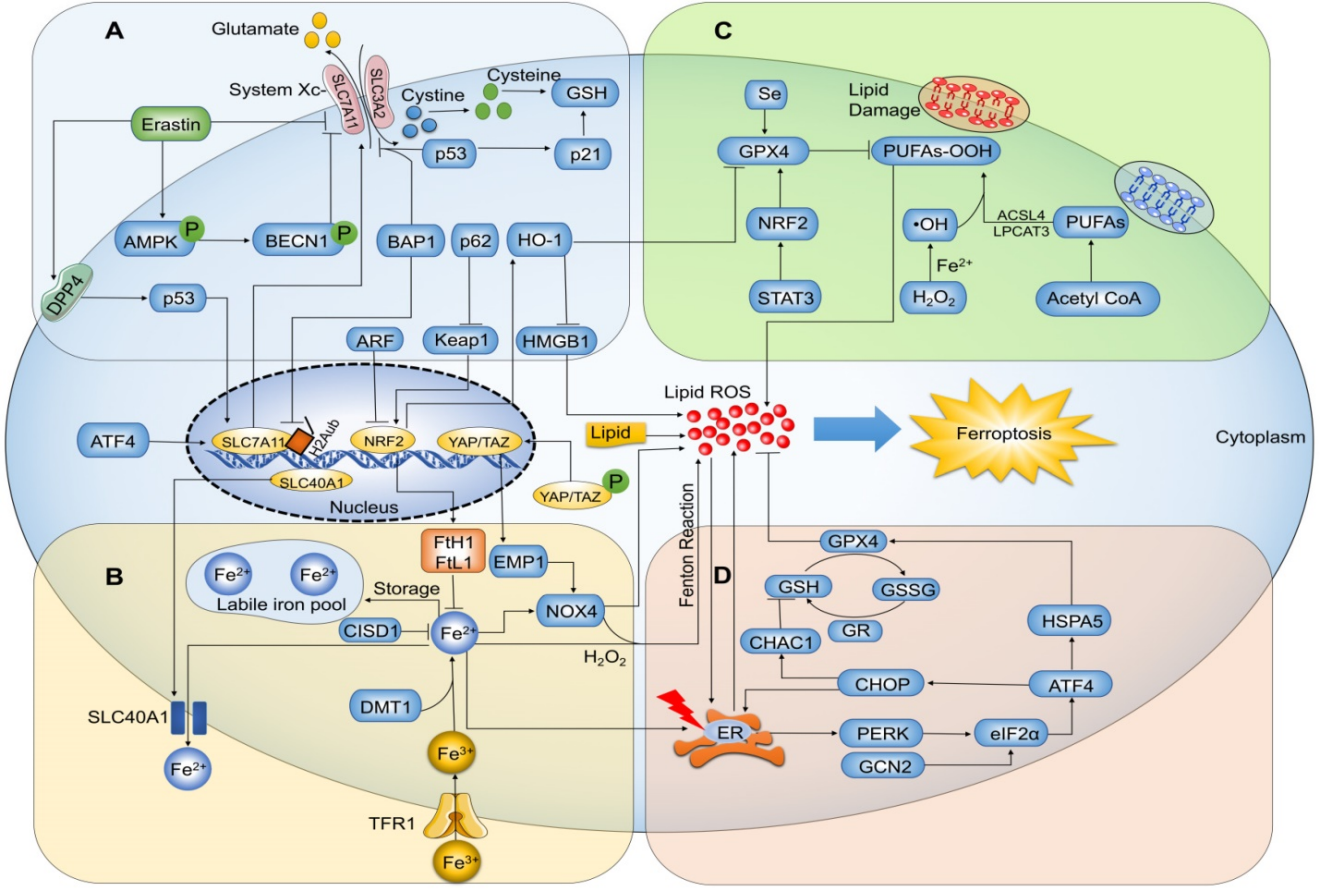
Ferroptosis-related signaling molecules and signaling pathways. (A) Glutamate exchanges for cystine in a 1:1 ratio through the cystine/glutamate antiporter system Xc-, and inhibition of system Xc- by its core part SLC7A11 induces ferroptosis. (B) Ferric iron (Fe^3+^) bound to transferrin enters cells via membrane protein transferrin receptor 1 (TFR1) and localizes in endosomes, wherein the ferrireductase activity of STEAP3 reduces Fe^3+^ to redox-active iron (Fe^2+^). Finally, divalent metal transporter 1 (DMT1) releases Fe^2+^ from endosomes into a labile iron pool in the cytoplasm. In general, excess iron is stored in ferritin with ferritin heavy chain 1 (FtH1) and ferritin light chain 1 (FtL1). Under the action of H_2_O_2_, Fe^2+^ catalyzes the production of hydroxyl radical (HO∙) by Fenton reaction, triggering a chain reaction of radical lipid peroxidation and eventually leads to ferroptosis. (C) Ferroptosis is trigged by peroxidation (-OOH) of polyunsaturated fatty acids (PUFAs) and aberrant accumulation of lipid reactive oxygen species (ROS), resulting in membrane destabilization and rupture. Acyl-CoA synthetase long-chain family member 4 (ACSL4) and lysophosphatidylcholine acyltransferase 3 (LPCAT3) are necessary for ferroptosis to produce the target lipid pool containing arachidonic acid. GPX4 can hydrolyze lipid peroxides into non-toxic lipid alcohols (-OH). (D) Intracellular glutathione exists as oxidized glutathione (GSSG) and reduced glutathione (GSH); GPX4 requires GSH as a cofactor and reduces GSSG to GSH via glutathione reductase (GR). GPX4 inhibits the formation of Fe^2+^-dependent ROS by converting lipid hydroperoxides into lipid alcohols, and thus inhibits ferroptosis.

Ferroptosis is a consequence of increased ROS levels caused by elevated intracellular iron concentration, leading to lipid peroxidation and cell death 23 (**Figure 2**). Iron is a basic component of many enzymes involved in DNA synthesis, heme and iron-sulfur cluster synthesis, and metastasis. As such, it plays a vital role in numerous essential life processes 24. Free iron is directly linked to ferroptosis because it promotes ROS production via Fenton reactions that cause lipid peroxidation 25. Ferric iron (Fe^3+^) bound to transferrin is the main form of circulating iron, which enters cells through membrane protein transferrin receptor 1 (TFR1) and localizes in endosomes, wherein the ferrireductase activity of STEAP3 reduces Fe^3+^ to ferrous iron (Fe^2+^). Finally, the divalent metal transporter 1 (DMT1) releases Fe^2+^ from endosomes into a labile iron pool in the cytoplasm. In general, excess iron is stored in ferritin with ferritin heavy chain 1 (FtH1) and ferritin light chain 1 (FtL1) 26. Studies have confirmed that ferroptosis-sensitive cells with mutations in Ras display increased expression of TFR1 and reduced expression of ferritin (FtL1 and FtH1) 27. The iron chelator deferoxamine (DFO) can significantly suppress ferroptosis to protect cells and alleviate ferroptosis-related diseases 28, 29. Since ferroptosis is mediated by intracellular iron overload, which is regulated by altered iron metabolism, a better understanding of its potential molecular and cellular mechanisms will likely provide novel approaches for ferroptosis regulation.

### Ferroptosis is regulated by mitochondrial iron metabolism

Mitochondria are the primary site of iron utilization, play a major role in regulating oxidative metabolism, and are also the main source of ROS 30. As the most common metal in mitochondria, iron plays an important role in regulating the physiological function of these organelles 31. Iron usually crosses both the outer and inner mitochondrial membranes to reach the matrix, where mitochondrial iron metabolism occurs. Iron transport across the inner mitochondrial membrane depends on the membrane transporter mitoferrin 1/2 (Mfrn1/2), the imbalance of which can lead to mitochondrial iron accumulation and oxidative injury 32. Mfrn1/2 damage has been observed in a variety of neurological diseases associated with ferroptosis 33. Moreover, mitochondrial iron metabolism is also regulated by the voltage-dependent anion channels, located in the outer mitochondrial membrane 34. Previous studies demonstrated that erastin-induced voltage-dependent anion channel 2/3 opening was correlated with mitochondrial iron accumulation and ferroptosis 35. It has also been reported that the accumulation of mitochondrial free iron aggravated erastin-mediated ferroptosis 36. Physiologically, mitochondrial free iron is strictly controlled by mitochondrial ferritin (FtMt), which can protect cells from the damage caused by mitochondrial ROS 37. Also, overexpression of FtMt can sequester iron in mitochondria and resist erastin-induced ferroptosis* in vivo* and *in vitro* 38.

### Ferroptosis is induced by cysteine deprivation

Amino acid metabolism is closely related to ferroptosis regulation, and cysteine is essential for ferroptosis regulation since its availability limits the biosynthesis of glutathione. Cysteine is oxidized to cystine, and an oxidized cysteine dimer linked by a disulfide bridge can be easily transported to mammalian cells as a natural analog of cysteine 2. In cells, cystine is reduced to cysteine, which is an indispensable substrate for the synthesis of biomolecules such as glutathione, proteins, and coenzyme A3 39, 40. As a heterodimeric cell surface amino acid antiporter, system Xc- is composed of the 12- transmembrane helix-containing transporter protein SLC7A11 (also known as xCT) linked by a disulfide bridge to the single-pass transmembrane regulatory protein SLC3A2 (4F2hc) 9. While glutamate can exchange for cystine in a 1:1 ratio via system Xc-, accumulation of extracellular glutamate induces ferroptosis by inhibiting system Xc- 8, 41. Importantly, erastin can directly inhibit system Xc- function resulting in significant depletion of intracellular glutathione 42. Cysteine in the culture medium is oxidized to cystine when molecular oxygen levels are high, and reductant levels are low. Therefore, SLC7A11 can protect cultured cells against cell death. Erastin disrupts cystine uptake by SLC7A11 and leads to cell death via inhibiting SLC7A11 activity, resulting in cysteine deficiency, glutathione depletion, and ferroptosis 41, 43. Thus, compounds or drugs that specifically influence the function of SLC7A11 and thereby modify ferroptosis have been explored *in vivo* and *in vitro* for their potential to treat various human diseases.

### Ferroptosis is induced by GSH depletion

Glutathione (γ-L-glutamyl-L-cysteinyl glycine) is a tripeptide containing a cysteine unit at its core that plays a key role in protecting against lipid peroxidation in ferroptosis by donating an electron to GPX4 41. Intracellular glutathione exists as reduced (GSH) and oxidized glutathione (GSSG), providing the main antioxidant buffer against oxidative stress 44. Studies have demonstrated that glutamate-cysteine ligase, the first rate-limiting enzyme in the two-step synthesis of glutathione, could be inhibited by buthionine-(S, R)-sulfoximine (BSO), leading to cell death. DFO and a-tocopherol could reverse this effect, but not the necroptosis inhibitor Necrostain-1 or the apoptosis inhibitor zVAD-fmk 9, 45, 46. Further studies confirmed that erastin induced ferroptosis by GSH down-regulation caused by depletion of intracellular cysteine, whereas p53-p21 signaling delayed ferroptosis by preserving GSH levels, and thereby had a pro-survival effect 47, 48. As a 12 kDa ubiquitous oxidoreductase, thioredoxin plays an essential role in the thioredoxin antioxidant system, composed of NADPH, thioredoxin, and thioredoxin reductase 49. In mammalian cells, thioredoxin and glutathione systems can cross-donate electrons and serve as backup systems for each other 50. Telorack et al. demonstrated that the thioredoxin systems could efficiently compensate deficiency in glutathione biosynthesis in keratinocytes to maintain antioxidant capacity 51. Therefore, inhibition of ferroptosis induced by glutathione depletion is an essential mechanism preventing oxidative stress and ferroptotic cell death.

### Ferroptosis is prevented by GPX4

GPX4, the only member of the GPX protein subfamily (GPX1-8), can reduce phospholipid hydrogen peroxide. It contains an efficient selenocysteine unit that can increase its peroxidase activity 52, 53. GPX4 inhibits the formation of Fe^2+^-dependent ROS by converting lipid hydroperoxides into lipid alcohols. Hence, inhibition of GPX4 leads to an increase of lipid ROS formation and lipid peroxidation, which induces ferroptosis 54. Evidence has revealed that GPX4 knockdown directly inhibits ferroptosis but does not affect other essential mechanisms 55. Consistent with this, lack of cysteine diminishes GSH synthesis and reduces GPX4 activity, eventually leading to ferroptosis 56, 57. RSL3, the first reported effective GPX4 inhibitor identified by chemical screening, has been widely used in the experimental induction of ferroptosis, especially in cancer chemotherapy 27. Notably, GPX4 ablation in adult mice resulted in embryonic lethality as evidenced by elevated 4-hydroxylnonenal (4-HNE), reduction in the activity of electron transport chain complexes I and IV, and decreased ATP production in mitochondria that eventually led to neuronal loss, suggesting that GPX4 has an essential role in mitochondrial integrity and neuronal survival 58. Another study confirmed that ferroptosis, rather than apoptosis, is the leading cause of embryonic lethality 45. Additionally, GPX4 is also involved in T cell immunity, as evidenced by GPX4 levels, which were lower in HIV-infected cell populations than in uninfected cells by using 75Se-labeled human Jurkat T cells 59. Another study revealed that GPX4-deficient T cells could rapidly accumulate membrane lipid peroxides accompanied by ferroptosis-mediated cell death rather than necroptosis 60. Since GPX4 can act as an important negative regulatory factor of ferroptosis by scavenging toxic intracellular lipid hydroperoxides, the development of drugs for the regulation of GPX4 is of great practical significance.

### Ferroptosis is induced by PUFAs

Excessive PUFA consumption, especially red and processed meat, has been associated with nutritional and environmental health hazards 61. High PUFA intake indicates an increased risk of ARDs, including cancers, type 2 diabetes, and CVDs, but the specific molecular mechanism remains unclear 62. Ferroptosis can be driven by excessive peroxidation of PUFAs, characterized by iron-catalyzed excessive peroxidation of PUFA-containing phospholipids 63. Although PUFAs can increase membrane fluidity and have beneficial effects on human health 64, exposure to excess substrates (iron or glutamate) can trigger enzyme-linked reactions by activating enzymes associated with the biosynthesis and remodeling of PUFAs in the cell membrane. These enzymes include lysophosphatidylcholine acyltransferase 3 (LPCAT3) and acyl-Co synthetase long-chain family member 4 (ACSL4), as well as enzymes that increase intracellular ROS, such as NADPH oxidase 65. PUFAs are oxidized by intracellular ROS and produce lipid peroxides that lead to ferroptosis 66. The destruction of cell membranes by lipid peroxidation can cause morphological changes, such as mitochondrial shrinkage and damage. Further, lipid peroxides decompose into reactive derivatives, including 4-HNE and malondialdehyde (MDA), which react with nucleic acids and proteins and thus destroy membrane integrity resulting in cell rupture 67. A recent study demonstrated that exogenous monounsaturated fatty acids could induce cellular resistance to ferroptosis and reduce the accumulation of lipid peroxides and oxidizable PUFAs 68. It is noteworthy that close interactions between PUFA metabolism, ROS, GPX4, and ferroptosis may ultimately determine cell survival or death. Hence, the regulatory mechanism of ferroptosis provides a new perspective for cell fate determination.

## Small-molecule modulators of ferroptosis

### Small molecule inducers of ferroptosis

Ferroptosis was initially defined through a group of small molecules (RAS selective lethal, RSL) that induce selective death of tumor cells carrying RAS mutations 69. Exploiting the high mutagenicity of the RAS family of small GTPases (NRAS, HRAS, and KRAS), Stockwell and colleagues identified two new RSL small molecules named RSL3 and erastin. Mechanistically, erastin mediates ferroptosis via inhibition of system Xc- and RSL3 functions by inhibiting GPX4; both inhibitors can cause ferroptosis without inducing morphological changes or biochemical processes similar to apoptosis 27, 70. Other studies have found that sorafenin 71 and glutamate 29 also induced ferroptosis via cystine uptake inhibition by the system Xc-. GSH inhibits ferroptosis by maintaining GPX4 function, essential to prevent harmful phospholipid oxidation. In this respect, a series of small molecules, including RSL3 55 and FIN56 72 was found to inactivate GPX4 and induce lipid peroxide accumulation directly, leading to ferroptosis. Other inducers of ferroptosis, such as buthionine sulfoximine 73 and cisplatin 74, may induce synthetic lethality similar to that caused by GSH depletion (**Table 1**).

### Small molecule inhibitors of ferroptosis

Most of the ferroptosis mechanisms were elucidated by the identification of cell death inhibitors, which are classified based on the ferroptosis-triggered lipid peroxidation mechanism. The disruption of redox homeostasis is one of the main causes of ferroptosis; both zileuton and N-acetylcysteine can protect cells from lipid peroxidation by down-regulating 5- lipoxygenase (LOX) 75, 76. Fat-soluble antioxidants, such as aryl alkylamine compounds Fer-1 and Lip-1, can specifically reduce ROS production to inhibit RSL-induced ferroptosis 77, 78. A recent study reported that SRS-16-86, a third-generation ferrostatin, has improved plasma and metabolic stability and acts as a ROS scavenger leading to ferroptosis blockage 79. Also, cellular iron overload is a significant feature in ferroptosis, and commonly used iron-chelating agents, such as deferasirox, deferiprone (DFP), or DFO, can reduce cell death caused by excessive free iron 80, 81. As a member of the long-chain fatty-acid-coenzyme A ligase family, ACSL4 is a key enzyme that regulates lipid composition. A previous report demonstrated that the expression of ACSL4 was remarkably down-regulated in ferroptosis-resistant cells, such as LNCaP and K562, and might serve as a contributor and biomarker of ferroptosis 82. Consistent with this observation, ACSL4 inhibitor rosiglitazone could pharmacologically modulate ACSL4 activity to suppress lipid peroxidation and prevent ferroptosis* in vivo* and *in vitro* 83.

### Regulation of ferroptosis by natural compounds

For decades, natural products were investigated as promising reagents for drug development. Many substances separated from plentiful natural resources are used to prevent and treat various diseases 84. Numerous roles of natural compounds in iron metabolism and homeostasis have been identified that are relevant to ferroptosis and treating ferroptosis-related diseases. Artemisia annua L. (Asteraceae) has been used in traditional Chinese medicine for 2000 years and is now used as the first-line treatment of malaria throughout Asia and Africa. Interestingly, research in recent years has shown that its biological activity is not limited to malaria treatment. Artemisin and its derivatives (*e.g.,* artesunate, artemisinin, and dihydroartemisinin) have been found to induce ferroptosis in cancer cells through ROS accumulation, an overload of lipid peroxides and iron, and triggering antioxidant stress responses 85-87. Llabani et al. 49 discovered that ferroptocide is a novel compound that can induce rapid ferroptosis and inhibit thioredoxin in primary cancer cells and immortalized cancer cell lines from patients.

Various natural compounds can induce ferroptosis, while others act as ferroptosis inhibitors to provide new tools for treating other diseases. Baicalein (from *Scutellaria baicalensis*), a selective inhibitor of arachidonate 12/15-LOX, has been identified through natural product library screening as a ferroptosis inhibitor 88, which exerts neuroprotection against post-traumatic epileptic seizures through ferroptosis suppression 89. Other natural compounds, such as gastrodin 29 and puerarin 90, are related to renal damage, glutamate-induced cell death, and heart failure through ferroptosis-associated mechanisms or inhibiting ferroptosis signaling pathways. Therefore, inhibition of ferroptotic death using natural products may provide new therapeutic strategies for ferroptosis-related diseases.

## Ferroptosis-related signaling molecules and signaling pathways

### ATF4 signaling

Activating transcription factor 4 (ATF4), a member of the cAMP response element-binding protein-2 family, participates in regulating multiple signaling pathways including autophagy, translation, oxidative stress, and inflammation, suggesting that it plays a multifaceted role in a variety of pathological processes 91. Under normal circumstances, ATF4 is constitutively expressed only at low concentrations. However, upon stimulation by microenvironmental stresses, such as anoxia and hypoxia and endoplasmic reticulum stress sensed by upstream eukaryotic translation initiation factor 2α (eIF2α) kinases, expression of ATF4 is elevated to influence development, metabolism, redox balance, and angiogenesis 92. Also, endoplasmic reticulum stress is related to the activation of ATF4-C/EBP homologous protein (CHOP) pathway, associated with ferroptosis-related diseases such as Burkitt's lymphoma and diabetes myocardial ischemia/reperfusion (I/R) injury 93, 94. A previous study showed that free uncharged tRNAs could trigger the GCN2-ATF4 axis to mediate a well-characterized transcriptional amino acid response under amino acid deprivation 95. As an amino acid deficiency sensor, GCN2-ATF4 signaling guides transcriptional control and protein synthesis and degradation 96. Further, degradation of GSH caused by ATF4 target gene CHAC1 enhanced cystine starvation-induced ferroptosis by the GCN2-eIF2α-ATF4 axis in human triple-negative breast cancer cells 97. Moreover, heat shock 70 kDa protein 5 (HSPA5), considered to be a molecular chaperone mediating endoplasmic reticulum unfolding of proteins, has been shown to negatively regulate ferroptosis by preventing GPX4 degradation 98. In glioma cells, dihydroartemisinin induces HSPA5 expression by protein kinase R-like ER kinase (PERK), and up-regulates the activity of ATF4, resulting in increased GPX4 and ferroptosis inhibition 85. The role of the ATF4-HSPA5-GPX4 axis has also been verified in human pancreatic ductal adenocarcinoma cells in a negative feedback pathway, offering a promising therapeutic strategy for overcoming drug resistance in tumors 99. As a critical mediator of oxidative and metabolic homeostasis, ATF4 has a dual role in ferroptotic cell death via complex networks of signal regulation and control 100. Further studies are needed for clarification of the precise regulatory effects of ATF4 on ferroptosis.

### NOX4 signaling

NADPH oxidase (NOX) is a major enzyme that transfers electrons from NADPH to molecular oxygen and shuttles electrons across biological membranes to produce superoxide. To date, five NOX genes (NOX1-5) have been identified in the human genome. Abnormal expression of NOX4 affects cell proliferation and apoptosis and is responsible for a variety of pathological processes 101, 102. Previous reports identified NOX4 to be a novel source of mitochondrial oxidative stress in cardiac myocytes and macrophages. Knockdown of NOX4 inhibits intracellular ROS production, macrophage cytotoxicity, and mitochondrial and DNA damage, implicating NOX4 in oxidative stress-mediated cell injury 101, 103. In HF rats induced by aortic banding, Toll-like receptor 4 (TLR4) or NOX4 gene knockout can dramatically improve left ventricular remodeling and reduce cardiomyocyte death by suppressing autophagy and ferroptosis 104. Based on this evidence, pharmacological inhibition or knockdown of NOX4 may partially prevent ferroptosis-induced cell death. Remarkably, ferroptosis is triggered by pseudolaric acid B in glioma cells via activating NOX4 by intracellular Fe^2+^, resulting in the overproduction of lipid peroxides and H_2_O_2_ and can be abolished by DFO, suggesting a novel target for cancer treatment 105. In summary, NOX4 can mediate various signaling pathways that participate in the induction of ferroptosis, and pharmacological blockade or genetic inactivation of NOX4 may protect against cell death.

### BECN1 signaling

BECN1 is a key macroautophagy regulator that promotes the formation of autophagosomes 21. A recent report revealed a novel role of BECN1-SLC7A11 complex formation in ferroptosis regulation in cancer cells. Mechanistically, phosphorylation of BECN1 at Ser90/93/96 through AMP-activated protein kinase (AMPK) promotes ferroptosis by binding to SLC7A11 and directly blocking the activity of system Xc-, contributing to cancer cell death 106. BECN1-SLC7A11-mediated ferroptosis was also observed in SH-SY5Y neuroblastoma cells 107. Another study found that the BECN1 expression mediated by ELAV-like RNA binding protein 1 (ELAVL1) could also promote ferroptosis by inducing autophagy and ferritinophagy activation in hepatic stellate cells 108. These findings collectively indicate that BECN1 can regulate both ferroptosis and autophagy induction, but its specific regulatory mechanism and pathophysiological significance remain to be elucidated. In recent years, ferroptosis has been described as autophagy-dependent cell death under specific conditions, since the classic activators of ferroptosis, including erastin and RSL3, can increase autophagy flux in various cells. Excessive autophagy, especially NCOA4-facilitated ferritinophagy, STAT3-induced lysosomal membrane permeabilization, and BECN1-mediated system Xc- inhibition may promote ferroptotic cell death 109, 110. Therefore, it is essential to measure autophagic activity and flux during ferroptosis to better understand the process and function of autophagy-dependent ferroptosis.

### YAP/TAZ signaling

Transcriptional regulators, such as yes-associated protein (YAP), and transcriptional co-activators with PDZ-binding motif (TAZ), known as Hippo signaling cascade effectors, have attracted widespread attention due to their relevance to organ growth, tissue homeostasis, cell proliferation, and cancer. YAP/TAZ are sensors of structural and mechanical cues mediated by the cellular microenvironment, making them exploitable as therapeutic targets in cancer and regenerative medicine 111, 112. A recent study showed that E-cadherin suppresses ferroptosis by activating the intracellular NF2 and Hippo signaling pathway in epithelial cells, while antagonizing this signaling pathway enables YAP to promote ferroptosis, suggesting that the NF2-YAP axis is responsible for the cancer cells' response to ferroptosis-inducing therapy 113. Yang et al. demonstrated that the ferroptosis-promoting effect of TAZ was attributed to its ability to regulate ferroptotic cell death through the TAZ-ANGPTL4-NOX2 axis in epithelial ovarian cancer 114. Moreover, TAZ can also regulate membrane protein 1 and NOX4 levels, resulting in lipid peroxidation and ferroptosis in renal cell carcinoma 115. Together, these findings indicate that YAP/TAZ and Hippo pathway effectors play a novel role in lipid peroxidation by triggering ferroptosis and have therapeutic potential for epithelial ovarian cancer, renal cell carcinoma, and other TAZ-activated tumors, and might be exploited to modulate ferroptosis.

### NRF2 signaling

Nuclear factor erythroid 2-related factor 2 (NRF2), as a transcription factor, participates in the adaptive cellular response following exposure to oxidative and electrophilic stresses. NRF2 binds to antioxidant response elements and promotes a variety of antioxidant gene transcription 116. NRF2 is mainly complexed with Kelch-like ECH-associated protein 1 (Keap1) and CUL3E3 ubiquitin ligase to maintain its stability via ubiquitin 117. Mechanistically, NRF2 activation can promote iron storage, reduce iron uptake, limit ROS production, regulate SLC7A11 activity and thus regulate ferroptosis 118, 119. A recent study demonstrated that the NRF2-Keap1 pathway plays a critical role in cancer cell proliferation and lowering ferroptosis via up-regulating SLC7A11 and amplifying glutamate secretion 120. Expression of p62 also likely prevents the degradation of NRF2 and enhances subsequent NRF2 nuclear accumulation through Keap1 inactivation, leading to ferroptosis inhibition 121. On the other hand, alternative reading frame (ARF) tumor suppressor can regulate ferroptotic responses by directly inhibiting the transcriptional role of NRF2 and suppressing its target genes, including SLC7A11. Chen et al. found that ARF inhibited the SLC7A11-activating ability of NRF2, resulting in tumor suppression by inducing ferroptosis in p53 null cells 122. Shin et al. proposed that NRF2-antioxidant response element (ARE) pathway activation contributed to making head and neck cancer (HNC) cells refractory to GPX4 inhibition via a reduced labile iron pool, leading to ferroptosis resistance 123. Also, among the iron metabolism proteins associated with iron availability and ferroptosis, both ferritin and heme oxygenase 1 (HO-1) are affected by NRF2 10. A recent study reported that HO-1 knockout could promote ferroptosis induced by erastin in kidney cells and hepatocellular carcinoma 121. Cotreatment with erastin and acetaminophen decreased the expression of HO-1, whereas activation of NRF2 up-regulated HO-1, suggesting that acetaminophen sensitized ferroptosis by regulating the NRF2/HO-1 signaling axis 124. Hence, NRF2 can act as a key negative regulatory factor of ferroptosis in complex molecular signaling networks and plays a protective role in cell death.

### p53 signaling

The tumor suppressor gene p53 inhibits tumorigenesis by initiating apoptosis, cell cycle arrest, and senescence. Recent research has challenged this notion by demonstrating p53 to be a transcriptional repressor of SLC7A11 that can impair cysteine import and promote ferroptosis. Under specific conditions, p53 is activated by various stress stimuli and favors organismal homeostasis by additional mechanisms, including ferroptosis induction. Various signaling pathways participate in ferroptosis regulation by p53 105, 125, 126. For example, p53 can indirectly activate the ALOX12 function via SLC7A11, leading to tumor suppression through a distinct ferroptosis pathway 127. Interestingly, Wang et al. uncovered a previously unappreciated epigenetic mechanism of ferroptosis regulation in which p53 negatively regulates mono-ubiquitination of histone H2B on lys120 (known as an epigenetic mark) by promoting the nuclear translocation of the deubiquitinase USP7, and represses SLC7A11 expression 128. Furthermore, spermidine/spermine N1-acetyltransferase 1 (SAT1) is a transcription target of p53 and participates in ferroptosis regulation during tumor suppression. In brief, p53-mediated SAT1 activation contributes to ferroptotic responses, and elevated SAT1 expression results in lipid peroxidation and overexpression of 15-LOX, thereby sensitizing cells to undergo ferroptosis during ROS stress 129. Given the finding that multiple signaling regulators and pathways are involved in ferroptosis, regulating these important signaling molecules and their transduction pathways is of great significance for understanding the pathophysiology of ferroptosis (**Table 2**).

## Ferroptosis regulation in ARDs

A growing body of research suggests that ferroptosis contributes to the progression of ARDs, including neurodegenerative and cardiovascular diseases while blocking ferroptosis by pharmacological agents or gene manipulations can inhibit cell injury, prevent disease progression, and improve disease symptoms. It has been reported that treatment with Lip-1 was neuroprotective in vitamin E-deficient diet-fed GPX4BIKO mice 130, and Fer-1 could significantly inhibit lipid peroxidation and ferroptotic cell death in cellular models of Huntington's disease 77. Furthermore, inhibition of ferroptosis with DFO or NAC treatment significantly reduced iron abundance and the level of oxidative stress, as well as increased cardiomyocyte viability in rat neonatal cardiomyocytes 131 (**Table 3**).

### Neurodegenerative diseases

#### Alzheimer's disease (AD)

AD is one of the most common neurodegenerative diseases of the central nervous system, characterized by neurofibrillary tangles and amyloid-β (Aβ) plaques in the brain. It is mainly manifested as progressive cognitive dysfunction and impaired behavior in the clinic. With the acceleration in the global aging process, AD affects nearly 44 million people worldwide 132. Although the scientific community and governments are vigorously promoting the development of new drugs for AD, there is no specific cure at present; symptomatic treatment and delay in disease progression are the only available measures, presenting a major challenge. Abnormal and massive deposition of Aβ plaques is a major pathological mechanism for AD, and drugs that target Aβ are highly sought after. However, in recent years, drugs targeting the Aβ protein have often failed in clinical trials. Thus, attention is being focused on gaining new molecular insights into AD development and seeking new treatment strategies to slow the progress of the disease.

Iron is crucial for the healthy development of the brain as it is used in the synthesis of neurotransmitters, myelin production, myelination, neuronal development, and other cell functions. It is reported that iron levels are elevated in the brain of individuals suffering from clinical AD and contribute to disease progression 12. Iron accumulation leads to nerve cell damage in patients with AD, likely by potentiating GSH loss 133 and iron deposition results in lipid peroxidation in cells, causing ferroptotic cell death. A recent study demonstrated that following treatment with high dietary iron, expression levels of ferroptosis-related antioxidants, including SLC7A11, GPX4, and superoxide dismutase in the brain, were decreased in APP/PS1 mice (a transgenic mice model of AD), suggesting that iron-induced neuron loss might occur through ferroptosis 134. Therefore, chelating iron ions may have a therapeutic effect on AD by inhibiting ferroptosis. Notably, the mitochondrial iron storage protein FtMt could prevent mitochondria from iron-induced oxidative injury, and FtMt knockout significantly aggravated the learning and memory impairment in an AD mouse model 135. Huang et al. reported that Mfrn1 knockdown decreased mitochondrial iron and ROS levels, thus delaying the disease progression in Alzheimer model of C. elegans, characterized by the reduction of paralysis rate and the extension of lifespans 136. Also, the iron chelator DFO had therapeutic effects on patients with AD 137. Compared to the control group, DFO treatment at a low dose could slow the clinical progression of AD-related dementia compared with the control group. Moreover, multi-target iron chelators HLA-20 ([5-(4-propargylpiperazin-1-ylmethyl)-8-hydroxyquinoline]) and M30 ([5-(N-methylN-propargylaminomethyl)-8-hydroxyquinoline]) also had potential therapeutic effects on sporadic AD 138. The iron-chelating action of α-lipoic acid could also suppress ROS production and increase GPX4 and SLC7A11 expressions in P301S mice, suggesting that α-lipoic acid treatment might enhance neuronal survival by regulating ferroptosis 139.

Many recent studies have revealed that some compounds with anti-AD effects have an inhibitory effect on neuronal loss related to ferroptosis. For example, the chalcone derivative 14a-c exhibited a potent anti-ferroptotic cell death activity against RSL3- or erastin-induced ferroptosis by inhibiting lipid peroxidation 140. Similarly, 7-O-cinnamoyltaxifolin and 7-O-feruloyltaxifolin inhibited ferroptosis induced by RSL3, and the novel oxindole compound GIF-0726-r also prevented ferroptosis induced by erastin in HT22 cells, a murine hippocampal neuronal cell line 141, 142. These findings indicated that natural product hybrids with suppressive effects on ferroptosis might serve as preventive neuroprotectants for treating neurodegenerative disorders such as AD. In another study, ferroptosis inhibitor Lip-1 treatment improved neurodegeneration in vitamin E-deficient diet-fed GPX4BIKO mice (a mouse model of conditionally deleting GPX4 in forebrain neurons) 130. Thus, although the precise effects and mechanisms of ferroptosis in the pathogenesis of AD remain still unclear, these results suggest that targeting ferroptosis could provide opportunities for developing novel AD treatments.

#### Parkinson's disease (PD)

PD is a progressive neurodegenerative disease characterized by dopaminergic neuronal death in the substantia nigra pars compacta (SNc), eventually resulting in rigidity, resting tremors, and other motor symptoms. Although the precise cause of dopaminergic neuronal loss is still unclear, it has been suggested that iron-induced dopaminergic degeneration is a key event in PD pathogenesis 143. The concentration of iron is elevated in the SNc of deceased and living PD patients and is considered a pathognomonic hallmark of the disease 144. Iron accumulation induces ferroptosis characteristics such as elevated hydroxyl radicals and lipid peroxidation, likely contributing to the oxidative injury of nigral dopaminergic neurons in PD 145. Therefore, chelating iron ions can inhibit ferroptosis and protect against neuronal injury in PD. Treatment with iron chelators can prevent dopaminergic neuronal loss in the SNc and rescue motor deficits in PD mouse model 146, 147. In the MPTP-induced parkinsonian phenotype, FtMt was shown to protect against neuronal damage by inhibiting cellular iron accumulation and subsequent oxidative stress 148. Importantly, a double-blind, placebo-controlled randomized clinical trial in PD patients has shown that DFP is safe and effective for PD treatment 147, suggesting that inhibition of ferroptosis by iron chelation might also provide therapeutic opportunities for PD patients.

Loss of glutathione in the substantia nigra is a major feature of PD. Depleted glutathione can cause nigral dopaminergic neuronal death and progressive motor imbalance 149. Clinical trials showed that glutathione level was restored following glutathione administration, indicating its mild therapeutic effect in PD patients 150, 151. GPX4, an important lipid repair enzyme in the inhibition of ferroptosis, was reduced in the SNc of deceased PD patients, while its up-regulation was associated with neuron density 152. Ablation of glutathione by depleting intracellular cysteine levels with erastin treatment, suppressing glutathione availability as a substrate for GPX4, could induce ferroptosis 46, 55. Additionally, GPX4 exerted a protective effect against neurodegeneration by regulating ferroptosis in the PD pathology 130. Depletion of GPX4 induced ferroptosis in motor neurons, leading to dramatic motor neuron degeneration and paralysis 153. Furthermore, GPX4 could prevent neuronal dysfunction and PD-like symptoms, and GPX4 loss in dopaminergic neurons induced anxiety behavior and diminished spontaneous locomotor activity 154.

Fer-1 derivatives, as iron chelators, are drug candidates for pharmacologically modulating ferroptosis 155. Administration of vitamin E, a ferroptosis inhibitor, can delay motor neuron death and paralysis caused by GPX4 depletion. The ferroptosis inhibitor Fer-1 could inhibit 1-methyl-4-phenylpyridinium (MPP+)-induced dopaminergic neuroblastoma cell (SH-SY5Y) death* in vitro*, a widely used PD model 156. Recently, Do Van et al. showed that neurotoxins, including erastin and MPP+, commonly used in PD models, could induce ferroptosis in LUHMES cells, a human neuronal precursor cell line 155. Furthermore, pre-treatment with ferroptosis inhibitors, including Fer-1 and the iron chelator DFP prevented the toxicity of glutathione depletion in LUHMES cells and MPTP toxicity in mice. Overall, these results demonstrate that dopaminergic neuronal loss in PD may partially be due to ferroptosis, indicating that blocking ferroptosis may have a neuroprotective effect on PD.

#### Amyotrophic lateral sclerosis (ALS)

ALS is a devastating neurodegenerative disease caused by lower and upper motor neuron loss, resulting in progressive paralysis and death. Although its etiology is not fully understood, motor neuron death is considered one of the main causes. Therefore, unveiling the mechanism of motor neuron death and intervening against it may provide a treatment strategy for ALS. The dysregulation of iron metabolism has been shown to play a vital role in ALS pathophysiology 157. Serum iron and ferritin levels are higher in ALS patients than controls and are associated with lower survival rates in ALS patients. Similar phenomena are observed in animal models of ALS. In a transgenic mouse model of ALS with G37R mutation in superoxide dismutase 1 (SOD1 G37R), iron levels were elevated in ventral motor neurons and glia. After treatment with the iron-selective chelator salicylaldehyde isonicotinoyl hydrazine, the lifespan of SOD1 G37R mice could be extended, spinal motor neuron survival was up-regulated and motor function improved 158. Similarly, iron accumulated in the spinal cords of SOD1 G93A-transgenic mice, another ALS model 157. More importantly, treatment with iron chelators, including M30 and VK-28, could delay the disease onset, extend the life of G93A-SOD1 ALS mice, and mitigate motor neuron damage 159, 160. Recently, a phase III clinical trial identified four biomarkers closely related to ferroptosis 161. Thus, pharmacological intervention with iron chelators (and ferroptosis inhibitors) can significantly improve the disease symptoms in animal models of ALS. Although the specific mechanism relating ferroptosis to ALS is unknown, these studies indicate that blocking ferroptosis may be a potential treatment for ALS.

#### Multiple sclerosis (MS)

MS is an autoimmune disease characterized by inflammatory demyelination of the central nervous system, targeting oligodendrocytes and myelin. Although the pathogenesis of MS is still unknown, new insights suggest that oligodendrocyte loss is one of the key pathophysiological events 162. Several studies have revealed that abnormal iron metabolism and the resulting cytotoxicity contribute to neurodegeneration and hence the pathogenesis and progression of MS 163, 164. Recent histological and magnetic resonance imaging (MRI) results have shown a high concentration of iron in the brains of MS patients and in an experimental autoimmune encephalomyelitis (EAE) animal model of MS, especially in oligodendrocytes 163-165. Accumulation of iron contributes to progressive axonal degeneration in MS through increased ROS production and the promotion of iron-mediated oxidative damage 163.

Following oxidative stress, lipid peroxidation and free radicals play an essential role in MS pathogenesis. Iron overload and lipid peroxidation have been observed in MS and EAE, indicating that ferroptosis may occur in MS. GPX4 levels were decreased in MS brains and EAE spinal cords, and GCLc, SLC7A11, and GSH levels were also significantly decreased in EAE mice relative to controls. The levels of lipid peroxidation products, MDA and 4-HNE, were also increased in EAE mice compared with controls and so was the proportion of damaged mitochondria with irregular matrices, disrupted membranes, and degenerated cristae 166. Furthermore, the chelation of iron using DFO and DFP (also a ferroptosis inhibitor) reduced the severity of EAE 167, 168. These findings indicate that neuronal damage of EAE shares the common characteristic features of ferroptosis, identifying it as a potential therapeutic target for MS progression.

#### Huntington's disease (HD)

HD, a neurodegenerative disorder, is caused by an abnormal repetition of the CAG trinucleotide sequence in the huntingtin gene, characterized by motor, behavioral, and cognitive dysfunction 169. The disease has characteristic features with selective loss of medium-spiny neurons and the formation of intraneuronal protein aggregates. Despite our understanding of HD's genetics, the precise mechanisms of neuronal death are still not completely understood. Hence, there is no effective intervention available to prevent or delay the development of HD. Iron dysregulation and its accumulation in cellular and subcellular sites of the brain are also implicated in HD pathogenesis. Previous studies reported a significant increase in iron levels in HD patients' basal ganglia that occurs early in the disease process 170. MRI and quantitative susceptibility mapping revealed excessive iron deposition in the occipital cortex, globus pallidum, and putamen in HD patients 171-173. Ferritin iron and ferroportin accumulation in striatum and cortex was also observed in HD patients compared with healthy controls 174, 175. Intraventricular administration of DFO or oral DFP (iron-selective chelation) relieved HD symptoms in an R6/2 mouse model 175, 176. Furthermore, decreased GSH levels occur in HD patients and HD mice induced by 3-nitropropionic acid (3-NP) and, and supplementing with cystamine or cysteamine could restrain 3-NP-induced HD striatal neuronal death by up-regulating GSH levels 177, 178. Besides, extensive lipid peroxidation was observed in HD patients and R6/2 and mN90Q73 HD mouse models 77, 178, 179. More importantly, Fer-1 treatment significantly inhibited lipid peroxidation and iron-induced cell death in cellular models of HD 77. Taken together, these findings suggest that ferroptosis might play a deleterious role in HD development, and inhibiting ferroptosis may provide an important strategy for the treatment of HD.

#### Stroke

Stroke has become one of the most common causes of morbidity and mortality worldwide and is the leading cause of disability. The incidence of stroke increases with age, doubling each decade after 45, with more than 70% of strokes occurring in individuals above 65. At present, the clinical treatment of stroke is still limited to intervention measures to restore blood flow by drug or mechanical thrombolysis with limited success, and there are no effective measures to protect the brain from ischemic cell death 180. Research on brain injury after stroke has mainly focused on excitotoxicity, inflammation, oxidative stress, and apoptosis 181. Oxidative stress has a crucial role in neuropathological lesions, and abundant non-heme iron in the brain triggers membrane lipid peroxidation via Fenton chemistry, leading to brain edema, mitochondrial damage, and functional disorders. Notably, oxidative injury has become a key index for evaluating I/R-induced neuronal injury. However, it is difficult to translate these benefits to the clinic. Therefore, clarification of protective mechanisms and the development of optimized pharmacological blocking agents are imperative 182.

Accumulating evidence revealed that ferroptosis contributes to stroke 183 and its inhibition can significantly ameliorate the disease severity and improve functional recovery. Tuo et al. reported that tau-mediated iron export could protect against ferroptotic injury after ischemic stroke 184, and carvacrol increased GPX4, thereby inhibiting ferroptosis from protecting against hippocampal neuron I/R injury after ischemic stroke in gerbils 182. Ferroptosis also induced neuronal death after hemorrhagic stroke 185 and a single dose of Se delivered into the brain could drive the expression of GPX4, protect neurons, and improve behavior in a hemorrhagic stroke model 186. Also, N-acetylcysteine (NAC), a precursor of GSH, targeted ALOX5-derived toxic arachidonic acid and synergistically acted with prostaglandin E2 to inhibit ferroptosis and improve the prognosis of mice after hemorrhagic stroke 187. Therefore, modulators of ferroptosis are potential pharmacological targets of stroke.

### Cardiovascular diseases

#### Cardiomyopathy

Cardiomyopathy is a heterogeneous group of myocardial diseases correlated with structural and functional abnormalities caused by the heart's abnormal mechanical and electrical activity, characterized by inappropriate ventricular hypertrophy or dilatation. Severe cardiomyopathy can cause cardiovascular death or progressive HF 188. Cell loss caused by terminally differentiated cardiomyocyte death is an important cause of cardiomyopathy. Different forms of cell death related to cell loss, such as autophagy, apoptosis, and necrosis, have been confirmed in myocardial injury 189.

The role of ferroptosis in myocardial pathology has also been investigated in recent years. Iron homeostasis plays a critical role in myocardial injury, and an iron overload cardiomyopathy is caused by the accumulation of iron in the myocardium 190. Moreover, myocardial hemorrhage can contribute to iron deposition in cardiac tissue, resulting in excessive ROS production, triggering pathological events such as inflammation 191. Doxorubicin has high cardiotoxicity, limiting its clinical application, and iron chelators exert cardioprotective effects against this cardiotoxicity 192. Ferroptosis, characterized by altered iron status, is also associated with cardiac oxidative stress during cardiac dysfunction. Studies demonstrated that ferroptosis was associated with diabetic myocardial I/R injury, and its inhibition could alleviate the injury 94. Moreover, the activities of glutathione peroxidase and SOD were decreased in myocardial tissues of diabetic cardiomyopathy rats, while the level of MDA was increased, and inhibition of these changes could protect against oxidative stress and inflammation in myocardial tissue 193. Another study showed that a lipid kinase ENPP2 involved in lipid metabolism in cardiomyocytes could protect these cells against erastin-induced ferroptosis 194. Results from Fang et al. strongly supported the idea that ferroptosis may serve as a target for the prevention of cardiomyopathy, and pharmacologically blocking ferroptosis and iron chelation therapy may provide a new strategy for the treatment of fatal heart disease 195.

#### Heart failure (HF)

Heart failure (HF) is a pathological condition in which the heart fails to pump enough blood to meet the body's need. It can be induced by many reasons, including myocytes' loss caused by cell death during the final stage of CVD. Under hemodynamic stress, such as hypertension or myocardial infarction, compensatory cardiomyocytes lead to myocardial hypertrophy, and if left uncontrolled, this hypertrophic response culminates in ventricular dilatation and progressive cell loss, eventually developing into HF 90. A previous study 131 demonstrated a significant reduction in FtH in an *in vivo* mouse model of HF and showed that iron deposition and the resulting increase in oxidative stress in hearts after myocardial infarction contributes to cardiomyocyte death. Moreover, treatment with DFO or NAC could significantly decrease the abundance of iron and the level of oxidative stress, as well as increase the viability in rat neonatal cardiomyocytes harboring an adenoviral vector expressing short hairpin RNA targeted to FtH 131. Lapenna et al. demonstrated that in contrast to young adult rabbits, aged rabbits possessed higher levels of redox-active catalytic low molecular weight iron, coupled with greater lipid and protein oxidation in the heart tissue. DFO administration could reduce H_2_O_2_/iron (Fenton reaction)-dependent damage in perfused hearts of aged rats but not of young adult rats. This suggests that iron status may be responsible for cardiac oxidative stress and hemodynamic dysfunction 196.

Evidence supports the idea that diabetic patients with HF display abnormal myocardial iron status, and DFO could alleviate coronary microvascular adaptation by inhibiting iron-catalyzed oxidative reactions 197. Interestingly, in HF mice induced by hypobaric hypoxia, treatment with two novel nitronyl nitroxide radicals could reduce oxidant stress via free radical scavenging activity, resulting in increased SOD activity, catalase, and GSH-Px, and reducing MDA and ROS 198. Liu and colleagues reported that ferroptosis was directly involved in HF, as demonstrated by an elevated labile iron pool and lipid peroxide levels in the HF rat model. Additionally, ferrastin-1 could reverse the decrease in erastin-induced cell viability, while the TLR4/NOX4 pathway promoted myocyte death by ferroptosis and autophagy during HF 90, 104. To sum up, these findings indicate that ferroptosis is associated with HF pathology and targeting ferroptosis may provide a novel anti-HF strategy.

#### Myocardial infarction (MI)

MI, known as heart attack, is an irreversible heart muscle death following a prolonged lack of oxygen/ischemia supply. MI is the most common cause of death worldwide, and elucidation of the underlying mechanisms represents a major opportunity and challenge for prevention and treatment 199. Iron deposition in peri-infarcted and non-infarcted areas has been observed in MI mice following the left coronary artery ligation 131. One study demonstrated that both ferroptosis inducers (*e.g.,* erastin and RSL3) and excess iron accelerated iron incorporation, lipid ROS generation, and triggered cell death in isolated adult mouse cardiomyocytes. These effects were inhibited by Fer-1, implicating the involvement of ferroptosis 200. The increase in ROS was attributed to reduced activity of antioxidant enzymes SOD, GPX1, and catalase following MI 201. Interestingly, supplementation with the cellular antioxidant GSH could enhance myocardial resistance to I/R *in vivo* and protect the intact heart against oxidative damage, suggesting that oxidative stress was involved in cardiac tissue injury and cardiomyocyte death 202. Furthermore, Park et al. proposed that MI could induce the reduction of GPX4, which may sensitize cardiac cells to ferroptosis under low GSH conditions 203. A recent mechanistic study showed that BTB domain and CNC homolog 1 (BACH1), as a regulator of iron and heme metabolism, could promote ferroptosis by coordinating transcriptional regulation of GSH and labile iron metabolism; BACH1^-/-^ mice were more resistant to MI than wild-type mice, and DFO could relieve the severity of ischemic injury 28. Thus, preventing ferroptosis may provide a new therapy for patients with MI.

### Other age-related diseases

#### Chronic obstructive pulmonary disease (COPD)

COPD is a group of chronic lung disorders, characterized by a slowly progressive irreversible bronchial obstruction. Its main pathological manifestation is pulmonary emphysema, and it often occurs in the elderly. COPD is mainly caused by cigarette smoke (CS), and without a curative treatment, it has become a leading cause of premature death in industrialized countries 204. Thompson et al. demonstrated that iron concentration was increased by smoking in bronchoalveolar lavage fluid and alveolar macrophages, resulting in disruption of iron homeostasis 205. The elevated iron concentrations in the lungs increased the risk of pulmonary injury 206. DeMeo's team identified an important susceptibility gene for COPD, an iron-responsive element-binding protein that could up-regulate mitochondrial iron loading in association with CS-induced inflammation and lung injury 207, 208. Interestingly, as a key risk factor of COPD, CS could trigger iron-catalyzed oxidative stress and lead to lung injury, suggesting that oxidant/antioxidant balance plays a critical role in COPD 209. A recent report demonstrated that CS could induce epithelial cell ferroptosis in COPD, indicating accumulated labile iron and enhanced lipid peroxidation, which could be eliminated by GPX4 knockout or Fer-1 treatment 210. The GSH-based antioxidant protection system plays a key role in oxidant/antioxidant imbalance in patients with COPD, and GSH-Px makes an important contribution to maintain lung function 138, 211. A clinical study on COPD patients confirmed that doxycycline treatment could dramatically decrease lipid hydroperoxides and overall oxidative stress while increasing GSH-Px, GSH, and total nitrite antioxidant capacity, thereby improving lung function 212. Given the important roles of iron homeostasis and lipid peroxidation in COPD, targeting ferroptosis might provide a novel opportunity for COPD treatment.

#### Diabetes mellitus (DM)

DM, a chronic metabolic and degenerative disease is characterized by hyperglycemia due to defective insulin secretion or insulin dysfunction 213. Dysfunctional islet β-cell secretion and programmed cell death are two associated pathological processes 214, 215. Islet β-cell death plays a crucial role in the occurrence and development of type 2 DM (T2DM) and suppressing islet β-cell death is a challenging clinical problem. Iron overload is an important factor leading to the deterioration of diabetes 216. Circulating iron and ferritin levels, a biomarker for increased body iron stores, are significantly elevated in patients with T2DM 217, 218. Furthermore, excess free reactive Fe^2+^ can catalyze ROS formation through the Fenton reaction, which induces oxidative stress 218. Plasma levels of enzymes, such as GSH and SOD, and H_2_O_2_ concentrations are reduced in diabetic patients and animal models 219-221. Additionally, blood levels of lipid peroxidation and MDA are higher in diabetic patients than in healthy individuals 220, 222. It has been demonstrated that high glucose could increase MDA levels and reduce SOD and GPX4 activities in SRA01/04 cells 223. Moreover, the functional variant GPX4 (rs713041) regulates the risk of complications in patients with type 1 diabetes 224. The GPX4 protein abundance was decreased in the DM rat myocardial tissue compared with normal rats. Inhibition of ferroptosis using Fer-1 could reduce DM myocardial I/R injury *in vivo* and cell injury *in vitro* 94. Also, ferroptosis-inducing agents such as RSL3 and erastin could induce human islet death and compromise islet function *in vitro*, which could be ameliorated by pre-treatment of islets with Fer-1 or DFO 225.

Ferroptosis is also related to arsenic-induced islet β cell dysfunction, and Fer-1 can suppress NaAsO_2_-induced ferroptotic islet β cell death and pancreatic dysfunction by inhibiting the mitochondrial ROS-autophagy-ferritin pathway 226. These findings collectively suggest that iron imbalance, oxidative stress, and lipid peroxidation often occur in diabetic patients, resulting in ferroptosis and consequently exacerbating pancreatic function loss, indicating that ferroptosis blockade may provide a potential therapeutic strategy for DM. Nevertheless, further research is required to clarify the role of ferroptosis inhibitors in DM animal models and accurately define the specific biological effects of ferroptosis in this age-related disorder *in vitro* and *in vivo*.

## Concluding remarks

Since the discovery of ferroptosis, researchers have mainly focused on tumor prevention and treatment. However, given our expanding understanding of the impact of ferroptosis, its role in other age-related diseases has recently received much attention. Human aging is accompanied by a general decline in physiological functions, especially during the later stages. Consequently, there is an increase in the incidence of neurodegenerative diseases, CVDs, and other ARDs. Depleted GPX4 and GSH, elevated iron, and excessive lipid peroxidation are common features in ferroptosis and ARDs. Accumulating evidence has demonstrated that cells that undergo ferroptosis could secrete factors that strongly activate the innate immune system, leading to lipid peroxidation, the root cause of tissue damage and organ failure. This is especially true for neurodegenerative disorders, CVDs, and diabetes, where ferroptosis may underlie neuronal loss and damage to cardiomyocytes and β-cells correlated with these diseases.

Ferroptosis is likely to be a major cause of degenerative diseases, but it is not known whether the pathological mechanisms and signaling pathways in animal models closely resemble those in human patients. Although the regulatory mechanisms and molecular pathways in ferroptosis have been extensively explored using *in vivo* and *in vitro* disease models, strong evidence for ferroptosis in human cells and human autopsy tissues is still lacking. Furthermore, ferroptosis simply provides a connection between the phenotype of basic organ dysfunction and the observed accumulation of lipid peroxidation products in human pathology, but the mechanism by which ferroptosis regulates cell and tissue degeneration is still unclear. Previous results have shown that iron accumulates in aging tissues 227; however, whether ferroptosis is related to cell senescence and tissue aging in ARDs needs further investigation. Despite intriguing questions, there are no clinical trials to directly investigate the effects of ferroptosis-specific inhibitors or activators in age-related degenerative diseases. Therefore, in future studies, targeting ferroptosis as a potential strategy for treating ARDs is clearly promising.

Numerous studies have provided insights into the mechanisms and factors associated with the regulation of ARDs by ferroptosis. In this context, the development of antibodies and drugs against ferroptosis might benefit patients with a broad spectrum of ARD-related diseases. Research in this field is still in its infancy, and much work is needed to elucidate the detailed processes and mechanisms through which ferroptosis participates in ARDs. Future work is expected to provide novel therapeutic strategies for preventing, controlling, and treating ARDs.

## Figures and Tables

**Table 1:** Small molecule regulators of ferroptosis

Regulators	Compounds	Structures	Targets	Model systems
Inducers	Erastin	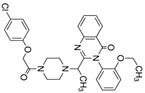	System Xc-	B16, HT-1080 228, HT-1080, 143B, BJeLR, Calu-1 8, Islets 225, MEF, A2780 74.
	Sorafenib	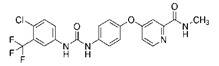	System Xc-	HepG2, Hep3B, Huh7 8, Huh7, PLC/PRF5, ACHN, BxPC-3, Caki-1, HCT116, SK-MEL-3, HT-29, NCI-H460, PANC-1 71.
	Sulfasalazine	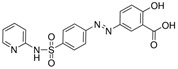	System Xc-	BJeLR, HT-1080 8, B16, HT-1080 228.
	Glutamate	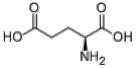	System Xc-	HT-1080 8, HT-22 29.
	(1S,3R)-RSL3	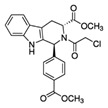	GPX4	B16, HT-1080 228, HT-1080, 143B 8, Islets 225, BJeLR, HT-1080 55.
	FIN56	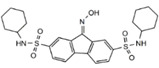	GPX4	BJeLR, HT-1080 72.
	FINO2	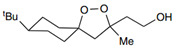	Iron	HT-1080 229.
	BAY11-7085	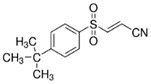	HO-1	MDA-MB-231, MDAMB-468, MCF-7, SKBR3, A549, HuH-7, DBTRG-05MG, SKOV3 230.
	t-BuOOH	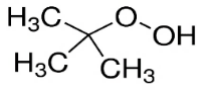	Lipid peroxidation	NIH3T3, ARPE-19 81.
	Buthionine Sulfoximine	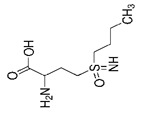	GSH	B16, HT-1080 228, HT-1080, BJeLR, DRD 55, HCT116, A549 73.
	Cisplatin	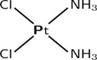	GSH	MEF, A2780 74.
Inhibitors	Ferrostatin-1	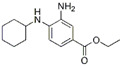	Lipid ROS	HT-22 29, ARPE-19 81, DAUDI, CA-46 93, HEK-293, HT22, MEF 69, HD brain-slice, oligodendrocytes, HT-1080 77, PTCs 80.
	Liproxstatin-1	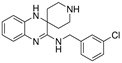	Lipid ROS	HT-22 29, HEK-293, HT22, MEF 69, DAUDI, CA-46 93, RILF 231, intestinal I/R 83, MOR 78.
	SRS16-86	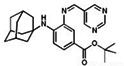	Lipid ROS	IRI 79.
Inhibitors	Vitamin E	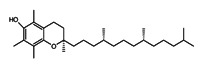	Lipid ROS	MiaPaCa-2 232.
	Trolox	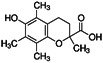	Lipid ROS	HCC1937, MDAMB-231, Hs 578T 97
	Deferasirox	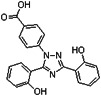	Iron	AMI 28.
	Deferoxamine	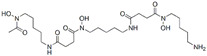	Iron	ARPE-19 81, HT-22 29, DAUDI, CA-46 93, PTCs 80, MEFs 28.
	Zileuton	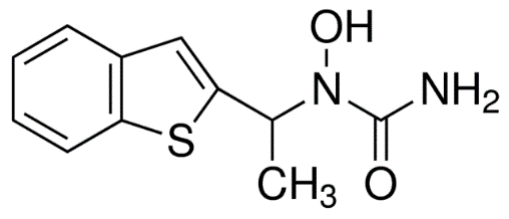	5-LOX	HT22 82.
	N-acetylcysteine	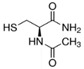	5-LOX	Neurons, IHC 187, PTCs 80.
	Rosiglitazone	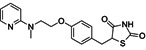	ACSL4	Intestinal I/R 83.

GPX4, glutathione peroxidase 4, HO-1, heme oxygenase 1, GSH, reduced glutathione, ROS, reactive oxygen species, 5-LOX, 5-lipoxygenase, ACSL4, acyl-Co synthetase long-chain family member 4, “NA”: not application.

**Table 2:** Ferroptosis-related signaling molecules and signaling pathways

Signaling molecules	Signaling pathways	Effects of signaling molecules	Mechanisms	References
ATF4	PERK-ATF4-HSPA5	Inhibition of ferroptosis in glioma cells.	PERK-upregulated ATF4 inducted HSPA5 expression and increased GPX4.	85, 99.
	GCN2-eIF2α-ATF4	Induction of ferroptosis in human triple negative breast cancer cells.	GCN2 activation increased eIF2α, ATF4 and CHAC1, CHAC1 degraded GSH.	97.
	ATF4-CHOP-CHAC1	Induction of ferroptosis in Burkitt's Lymphoma.	The ATF4-CHOP-CHAC1 axis degraded intracellular GSH and up-regulated CHAC1.	93.
	ATF4-SLC7A11	Inhibition of ferroptosis in human gliomas.	ATF4 activation elevated SLC7A11.	100, 233.
	ATF4-CHOP	Induction of ferroptosis in DIR injury.	The activation of ATF4-CHOP produced ERS and interacted with ROS in ferroptosis.	94.
NOX4	TAZ-EMP1-NOX4	Induction of ferroptosis in renal cell carcinoma.	TAZ up-regulated EMP1, EMP1 increased NOX4 and resulted lipid peroxidation.	115.
	TLR4-NOX4	Induction of ferroptosis in rats with heart failure.	TLR4 knock-down repressed NOX4, which inhibited cell loss.	104.
	Fe^2+^-NOX4-H_2_O_2_	Induction of ferroptosis in glioma cells.	Fe^2+^ activated NOX4 resulting in H_2_O_2_ and lipid peroxides overproduction.	105.
	EGFR-MAPK-NOX4/GPX4	Induction of ferroptosis in nonsmall-cell lung cancer cells.	Activated EGFR stimulated MAPK signaling, reduced GPX4 and induced NOX4.	234.
BECN1	AMPK-BECN1-SLC7A11	Induction of ferroptosis in tumor suppression.	AMPK-Mediated BECN1 phosphorylation blocked SLC7A11.	106, 107.
	ELAVL1/HuR-BECN1-autophagy	Induction of ferroptosis in hepatic stellate cells.	ELAVL1 triggered autophagy and promoted autophagic ferritin degradation by banding to the AREs of the BECN1 mRNA 3'-UTR.	108.
YAP/TAZ	E-cadherin-NF2-Hippo-YAP	Induction of ferroptosis in epithelial cells.	E-cadherin activated the intracellular NF2 and Hippo signaling pathway to suppress ferroptosis.	113.
	TAZ-ANGPTL4-NOX2	Induction of ferroptosis in epithelial ovarian cancer.	TAZ-regulated ANGPTL4 sensitized ferroptosis by activating NOX2.	114.
	TAZ-EMP1-NOX4	Induction of ferroptosis in renal cell carcinoma.	TAZ up-regulated EMP1, EMP1 increased NOX4 and resulted lipid peroxidation.	115.
NRF2	NRF2-TGF-β1	Inhibition of ferroptosis in lung fibrosis.	NRF2 signaling down-regulated TGF-β1 and balanced the ROS level.	231.
	NRF2-HO-1	Inhibition of ferroptosis in non-small-cell lung cancer.	NRF2 rescued HO-1 downregulation.	124.
	STAT3-NRF2-GPX4	Inhibition of ferroptosis in osteosarcoma cells.	Over-activation of STAT3/NRF2 increased GPX4 activity.	235.
	NRF2-Keap1	Inhibition of ferroptosis in primary malignant brain tumors.	NRF2-Keap1 signaling upregulated SLC7A11 and amplified glutamate secretion.	120.
	p62-Keap1-NRF2	Inhibition of ferroptosis in hepatocellular carcinoma cells.	p62 prevented the degradation of NRF2 and enhanced subsequent NRF2 nuclear accumulation via of Keap1 inactivation.	121.
	NRF2/p62-ARE	Resistance to ferroptosis in head and neck cancer.	p62-Keap1 interaction activated NRF2, increased ARE resulting in a decreased labile iron pool.	123.
	ARF-NRF2	Induction of ferroptosis in tumor suppression.	ARF inhibited NRF2 ability to activate its target genes SLC7A11.	122.
p53	p53-USP7-H2Bub1-SLC7A11	Sensitizing cells to erastin-induced ferroptosis.	p53 negatively regulated H2Bub1by promoting the nuclear translocation of the deubiquitinase USP7 and repressed the expression of SLC7A11.	128.
	p53-ALOX12	Induction of ferroptosis in tumor suppression.	p53 activated ALOX12 indirectly by transcriptional repression of SLC7A11.	127.
	p53-SLC7A11	Induction of ferroptosis in tumor suppression.	p53 repressed SLC7A11 transcription, reduced cystine uptake, and limited GSH.	105, 236, 237.
	SOCS1-p53	Induction of ferroptosis in tumor suppression.	SOCS1 activated p53 via both phosphorylation and stabilization.	238.
	p53-STAT1-ALOX15	Induction of ferroptosis in tumor suppression.	p53 directly activated SAT1, and increased the expression of ALOX15.	129.

ATF4, activating transcription factor 4; PERK, protein kinase R-like ER kinase; HSPA5, heat shock 70 kDa protein 5; eIF2α, translation initiation factor 2α; CHOP, C/EBP homologous protein; SLC7A11, solute carrier family 7 member 11; NOX4, NADPH oxidase 4; TAZ, transcriptional coactivator with PDZ-binding motif; EMP, epithelial membrane protein 1; TLR4, Toll-like receptor 4; EGFR, epidermal growth factor receptor; GPX4, glutathione peroxidase 4; AMPK, AMP activated protein kinase; ELAVL1/HuR, ELAV like RNA binding protein 1; YAP, yes-associated protein; NRF2, nuclear factor (erythroid-derived 2)-like 2; TGF-β1, transforming growth factor-β1; HO-1, heme oxygenase-1; STAT3, signal transducer and activator of transcription 3; Keap1, Kelch-like ECH associated protein 1; MAPK, mitogen activated protein kinase; ARE, antioxidant response elements; ARF, alternative reading frame; H2Bub1, monoubiquitination of histone H2B at lysine 120; ALOX12, arachidonate 12-lipoxygenase.

**Table 3:** Signs of ferroptosis and ferroptosis in age-related diseases

Diseases	Model systems	Biomarkers	Effects of blocking ferroptosis	References
**NDs**				
AD	P301S Tau transgenic mice	Iron, SOD1, GPX4, xCT, ROS, FPN1, TFR,	Tau phosphorylation↓, iron overload↓, lipid peroxidation↓, inflammation↓, learning ability↗, spatial memory↗.	139
	HDI-treated APP/PS1 mice	FPN, TFR, DMTI, ROS, mitochondria dysfunction	NA	134
	HT22 cells	GSH, xCT, GR, GCL, GST, ROS.	ROS accumulation↓, Ca^2+^ influx↓, oxidative stress-induced cell death↓.	133, 142
	SH-SY5Y cells	Lipid peroxidation	Aβ_1-42_ aggregation induced toxicity↓, lipid peroxidation↓.	140
	HT22 cells, BV-2 cells, AD mice model	GSH, NRF2	ATP loss↓, cell survival↑, neuroinflammation↓, short-term memory↗.	141
	GPX4BIKO mice	GPX4, lipid peroxidation	Neural protein NeuN↑, Synaptophysin↑, SNAP25↑, neurodegeneration↓, inflammation↓.	130
PD	LUHMES cells	Oxidative stress, ROS	New brain cells↑, oxidative stress↓, cell death↓.	239
	GPX4 knockout mice	GPX4, oxidative stress	NA	154
	SH-SY5Y cells	Lipid peroxidation	ROS/RNS↓, α-syn aggregation↓, cell death↓.	240, 241
	LUHMES cells, MPTP mice model, OSCs	SLC7A11, GPX4, GSH	MPTP's toxicity↓, dopaminergic neurons loss↓.	155
ALS	NSC-34 cells	Oxidative stress, ROS	New brain cells↑, oxidative stress↓, cell death↓.	239
	Plasma samples of patients	Lipid peroxidation, ferritin, iron	NA	161
	SN4741, N27 cells, primary cortical neurons	Lipid peroxidation, Fe^II^	Lipid peroxidation↓, lipid radicals↓, ferroptotic lethality↓, cell death↓.	242
MS	EAE, patients	GSH, GPX4, xCT, γ-glutamylcysteine ligase	NA	166, 243
	EAE	NA	Active EAE disease↓, T-cell function↓, inflammatory cell infiltrates↓, the clinical signs↓.	167, 168
HD	mN90Q73 HD mice	ROS	Healthy medium spiny neurons↑.	77
	HD (R6/2) transgenic mice, Human tissue samples, the striatal neurons	Lipid peroxidation	4-HNE adduct formation↓, ATP generation↗, mitochondrial morphology and function↗, mice lifespan↑.	179
	HD patients, HD animal model	Lipid peroxidation, GSH, SOD, CAT	NA	178
	R6/2 HD mice	TFR, FPN, IRPs, iron	Rota-rod endurance↗, lateral ventricles on the treated side↓.	175
Stroke	Hippocampal neurons, I/R gerbils	MDA, SOD1, CAT, TFR-1, GPX4, FPN1	Lipid peroxide↓, cell death↓.	182
	MCAO rats	DMT1, ROS, TFR1, SCL7A11, GPX4, MDA	Iron deposition↓, neurobehavioral scores↓, the numbers of Nissl bodies and visible nuclei↑.	183
	Cortical neurons, ICH mice and rats	GSH, ALOX5	Neutralizing toxic lipids↓, neuronal death↓, functional recovery↗.	187
	Focal cerebral ischemia model	Iron	Cognitive impairment↗, ongoing neuronal damage↓.	184
	ICH mice, primary cortical neurons, HT22	NA	Neuronal death↓, hemoglobin-and hemin-induced toxicity↓.	185
**CVDs**				
Cardiomyopathy	H9c2 cells, I/R rat model	ROS, GPX4, ACSL4, NRF2, MDA, SOD, Fe^2+^	Cardiomyocyte death↓, myocardial injury↓, the cardiac function of ischemic cardiomyopathy↗.	94, 194
	H9c2 cells, Nrf2^-/-^ mice, I/R mice model	MDA, NRF2, iron, oxidized lipids	Cardiac hypertrophy Anp, Bnp, and Myh7↓, cardiac function↗, mitochondrial function↗.	195
HF	H9c2 cells, aortic banding rats	GPX4, FtH1, iron, NOX4	Cell viability↑, mitochondrial atrophy↓, striated muscle arrangement↗.	90
	NOX4 knock-down aortic banding rats	GPX4, FtH1	Myocyte area noted↓, myocyte death↓.	104
MI	Cardiomyocytes, I/R mice model	ROS, iron, TFR1, ferritin	Cardiomyocyte death↓.	200
	MI mice model, H9c2 and C2C12 cells, NRVMs	MDA, GSH, GPX4, ACSL4, ROS	Myocardial cell death↓, lipid peroxidation↓.	203
	AMI mice model, MEFs	SLC7A11, GCLC, FtH1, FtL1, GSH	Cardiomyocyte death↓, the severity of AMI↓.	28
**Other ARDs**				
DM	DM and DIR model	GPX4, ACSL4, NRF2, MDA, SOD, Fe^2+^	The myocardial tissue lesions↓.	94
	MIN6 cells, NaAsO_2_-exposed rats	GSH, T-SOD, GPX4, MDA, ROS, COX2	Mitochondrial membrane potential↓, cytochrome c↓, MtROS↑.	226
COPD	HBECs, GPX4^+/-^ and GPX4 TG mice	GPX4, iron, ferritin	Lipid peroxidation↓, cell death↓, lung airspace enlargement↓, airway wall thickening↓.	210

ARDs, age-related diseases; NDs, neurodegenerative diseases; AD, Alzheimer's disease; PD, Parkinson disease; HD, Huntington's disease; ALS, amyotrophic lateral sclerosis; MS, multiple sclerosis; CVDs, cardiovascular diseases; HF, heart failure; MI, myocardial infarction; DM, diabetes mellitus; COPD, chronic obstructive pulmonary disease; “↑”: upregulation; “↓”: downregulation; “↗”: improving; “NA”: not application.
